# Robot-assisted vs. conventional MIDCAB: A propensity-matched analysis

**DOI:** 10.3389/fcvm.2022.943076

**Published:** 2022-08-30

**Authors:** Jan Gofus, Stepan Cerny, Youssef Shahin, Zdenek Sorm, Martin Vobornik, Petr Smolak, Ananya Sethi, Samuel Marcinov, Mikita Karalko, James Chek, Jan Harrer, Jan Vojacek, Marek Pojar

**Affiliations:** Department of Cardiac Surgery, Faculty of Medicine and University Hospital in Hradec Králové, Charles University, Hradec Králové, Czechia

**Keywords:** coronary artery bypass grafting, minimally invasive surgery, robotic surgery, internal thoracic artery, MIDCAB

## Abstract

**Background:**

Robotic assistance (RA) in the harvesting of internal thoracic artery during minimally invasive direct coronary artery bypass grafting (MIDCAB) provides several potential benefits for surgeon and patient in comparison with conventional MIDCAB. The two technical options have not been thoroughly compared in the literature yet. We aimed to perform this in our cohort with the use of propensity-score matching (PSM).

**Methods:**

This was a retrospective comparison of all consecutive patients undergoing conventional MIDCAB (2005–2021) and RA-MIDCAB (2018–2021) at our institution with the use of PSM with 27 preoperative covariates.

**Results:**

Throughout the study period 603 patients underwent conventional and 132 patients underwent RA-MIDCAB. One hundred and thirty matched pairs were selected for further comparison. PSM successfully eliminated all preoperative differences. Patients after RA-MIDCAB had lower 24 h blood loss post-operatively (300 vs. 450 ml, *p* = 0.002). They had shorter artificial ventilation time (6 vs. 7 h, *p* = 0.018) and hospital stay (6 vs. 8 days, *p* < 0.001). There was no difference in the risk of perioperative complications, short-term and mid-term mortality between the groups.

**Conclusions:**

RA-MIDCAB is an attractive alternative to conventional MIDCAB. It is associated with lower post-operative blood loss and potentially faster rehabilitation after surgery. The mortality and the risk of perioperative complications are comparable among the groups.

## Introduction

Surgical myocardial revascularization remains the standard of care in selected patients with coronary artery disease (1–3). As firstly described by Benetti in 1994, a single bypass of LITA to LAD may be safely performed *via* small left-anterior thoracotomy without the use of cardiopulmonary bypass, i.e., the minimally invasive direct coronary artery bypass grafting (MIDCAB) (4). There is a robust evidence that MIDCAB provides excellent short- and long-term results not only in patients with single-vessel disease, but also as a part of hybrid strategy in combination with percutaneous interventions in patients with multi-vessel disease (5–7).

With an advancement of robotic surgical technologies, the MIDCAB has been increasingly performed with the robotic assistance during LITA harvest (RA-MIDCAB) or even as a totally endoscopic procedure (TECAB) (8, 9). This strategy has been repeatedly compared with standard full sternotomy approach yielding superior short-term results (10–12). With the use of current robotic surgical technology, RA-MIDCAB could provide several potential benefits.

In the conventional MIDCAB, the LITA is harvested under direct vision with the use of special retractor, often with a need of longer skin incision, with an adjacent risk of rib fracture and a limited graft length in the end. In the RA-MIDCAB, the LITA harvest is performed under perfect visual conditions. The telemanipulation allows the surgeon to be more flexible and harvest a longer graft than in conventional settings. The thoracotomy for the distal anastomosis is smaller, there is less damage to the rib cage, leading to eventually faster rehabilitation and lower complication rate. However, a relevant direct comparison of conventional MIDCAB with RA-MIDCAB using the current robotic technology has not been performed yet (13, 14).

MIDCAB has been performed at our department for 25 years now, and since 2005 we have systematically recorded the perioperative data into a computer database. In 2018, a program of robotic cardiac surgery was initiated at our institution, RA-MIDCAB being the most important and the most frequent robotic procedure performed. In this analysis we aimed to provide a direct comparison of the two abovementioned techniques in terms of perioperative outcomes and long-term post-operative survival with the use of propensity-score matching to adjust for eventual confounders.

## Materials and methods

**Figure 1:**
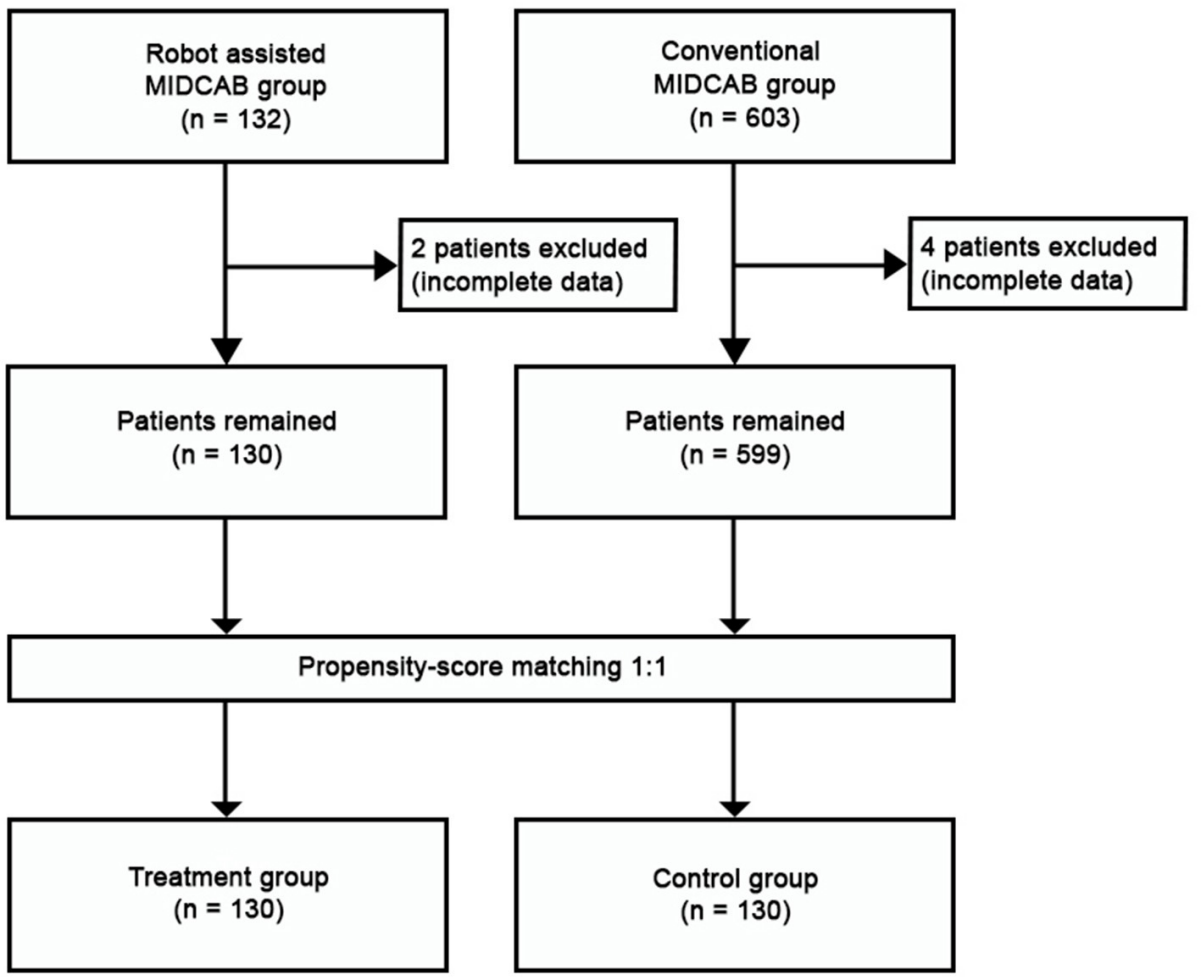
Study flowchart. MIDCAB, minimally invasive direct coronary artery bypass grafting.

This was a retrospective observational single-center study. All consecutive patients undergoing MIDCAB (since 2005) and RA-MIDCAB (since 2018) were included in the study. The learning curve was included. The perioperative data were extracted from patient in-hospital records and the long-term data on survival were provided by the Institute of health information and statistics of the Czech Republic. The closing date for patient inclusion and follow-up in both groups was December 31st, 2021. While the observed long-term outcomes were taken only from the compulsory national registry, we considered the follow-up 100% complete. The study was approved by institutional ethics committee (Ethics Committee at the University Hospital Hradec Kralove, Reference Number: 202202 P03). The patient informed consent was waived. The data underlying this study will be shared upon a reasonable request to the corresponding author. The study flowchart is visualized in Figure 1.

### Surgical technique

All patients were prepared in a supine position with their left chest elevated by ~30 degrees. After routine general anesthesia induction, a double-lumen endotracheal tube or bronchial blocker was used to decompress the left lung.

#### Conventional *MIDCAB*

A short (7–10 cm) left anterior thoracotomy was made at the fourth intercostal space. Care was taken to avoid injury of the mammary tissue while exposing the operative field. The LITA was harvested completely under direct vision with the help of specialized retractors. A rib spreader was inserted and the pericardium was incised. The target vessel was identified. The anastomosis of the LITA to LAD was performed through the incision with off-pump technique using standard stabilization devices (various brands have been used over the years, not recorded in our database).

#### RA-MIDCAB

The surgery started with an introduction of 3 ports in the anterior axillary line in the second, fourth, and sixth intercostal space and the da Vinci Xi robot system (Intuitive Surgical, Inc., Sunnyvale, CA, USA) was docked. A camera and 2 lateral arms with surgical instruments were introduced into the left pleural cavity. The LITA was harvested under continuous CO_2_ insufflation. The full length of LITA graft was harvested using both the low energy monopolar electrocautery spatula and the bipolar cautery forceps applied to the side branches. The LITA was harvested in a semi-skeletonized fashion with accompanying veins. Under endoscopic control, pericardial fat was removed, LAD was found and optimal anastomotic site was identified. An appropriate intercostal space for the construction of anastomosis was determined using a needle inserted through the chest wall. A minithoracotomy (5–6 cm) was performed. The off-pump anastomosis of LITA to LAD was performed on the beating heart under direct vision with the aid of suction stabilizer (Octopus Nuvo Tissue Stabilizer, Medtronic, Minneapolis, USA).

### Statistical analysis

All statistical analyses were performed with R (The R Foundation for Statistical Computing, Vienna, Austria, version 4.0.3) in RStudio (RStudio, Inc., Version 1.2.5042). The baseline characteristics of the two cohorts were compared with Wilcoxon rank sum test for continuous variables or with the two-tailed Fisher's exact test or Pearson's Chi-squared test for categorical variables. In Fisher's test for tables larger than 2 × 2 *p*-value was simulated using the Monte Carlo simulation, in 2 × 2 tables the exact *p*-value is reported. Multiple comparison correction was carried out using the Bonferroni correction.

Propensity score matching was performed with the MatchIt package version 4.0.0. The input covariates were the baseline characteristics of the cohorts and preoperative findings, total counts of covariates: sex, age, BMI, diabetes, hypertension, smoking status, renal failure, creatinine, dyslipidemia, heart rhythm, cerebral atherosclerosis (radiologically proven), peripheral atherosclerosis (radiologically proven), chronic obstructive pulmonary disease, history of stroke, Canadian Cardiovascular Society (CCS) angina class, New York Heart Association (NYHA) dyspnea class, history of infarction, previous cardiac surgery, history of percutaneous intervention, left-ventricular ejection fraction, severe heart valve disease, need of intravenous nitrates preoperatively, need of intravenous inotropes preoperatively, aspirin intake, intake of other antiaggregants, anticoagulants intake, and urgency of surgery.

Missing data were detected in the following covariates: hypertension, CCS class, NYHA class, previous cardiac surgery. The missingness of data was considered to be random and the amount of missing data was very low. Thus, cases containing missing data were removed before propensity matching and only complete cases were used for the analysis. The method used for matching was 1:1 matching of nearest neighbors without replacement. The outcomes of the treatment in the propensity score-matched cohorts were estimated using the two-tailed Mann–Whitney *U*-test for continuous variables or with the two-tailed Fisher's exact test or Pearson's Chi-squared test for categorical variables as described above, both with the Bonferroni correction. In all analyses, two-tailed *p*-values <0.05 were considered statistically significant.

The Kaplan-Meier survival analysis was performed using the R packages survival version 3.2-7 and survminer version 0.4.8. Statistical significance of differences between survival curves was determined with log-rank test. The survival estimates at pre-specified time-points were calculated together with their 95% confidence intervals (CI).

## Results

Throughout the study period, 603 patients underwent conventional MIDCAB and 132 patients underwent RA-MIDCAB at our institution (see Figure 1). Six patients (4 from MIDCAB group and 2 from RA-MIDCAB group) were excluded from the study pre-analysis due to incomplete dataset. Using the propensity-score matching, 130 pairs were found and compared. The matching successfully eliminated all preoperative differences between the groups (see Table 1). There was no 30-day or in-hospital post-operative mortality in either group post-matching. We observed no difference in the operation times between the groups despite including the learning curve of RA-MIDCAB. There were two conversions to median sternotomy in conventional group. The reason for conversion was extreme obesity in first patient, and right ventricular injury in second. In RA group there was a single conversion to median sternotomy due to right ventricular injury.

**Table 1:** Preoperative cohort characteristics pre- and post-matching.

	**Pre-matching**			**Post-matching**		
**Variable**	**Robot-assisted** **MIDCAB (*n* = 130)**	**Conventional MIDCAB (*n* = 599)**	***p*-value**	**Robot-assisted MIDCAB (*n* = 130)**	**Conventional MIDCAB (*n* = 130)**	***p*-value**
Female sex, *n* (%)	15 (12)	139 (23)	**0.015**	15 (12)	20 (15)	>0.9
Age (years), median (IQR)	66 (57, 73)	66 (58, 74)	0.6	66 (57, 73)	64 (55, 72)	>0.9
BMI (kg/m^2^), median (IQR)	28.4 (26.6, 31.3)	28.0 (25.5, 31.3)	0.5	28.4 (26.6, 31.3)	28.7 (25.7, 31.9)	>0.9
Diabetes, *n* (%):			0.7			>0.9
Diet	4 (3.1)	26 (4.3)		4 (3.1)	3 (2.3)	
OAD	24 (18)	117 (20)		24 (18)	26 (20)	
Insulin	18 (14)	60 (10)		18 (14)	14 (11)	
Hypertension, *n* (%)	110 (85)	470 (78)	0.2	110 (85)	114 (88)	>0.9
Smoking status, *n* (%):			0.12			>0.9
Non-smoker	39 (30)	245 (41)		39 (30)	47 (36)	
Exsmoker	48 (37)	205 (34)		48 (37)	47 (36)	
Smoker	43 (33)	149 (25)		43 (33)	36 (28)	
Renal failure, *n* (%)	3 (2.3)	32 (5.3)	0.3	3 (2.3)	3 (2.3)	>0.9
Dyslipidemia, *n* (%)	108 (83)	388 (65)	**<0.001**	108 (83)	112 (86)	>0.9
Heart Rhythm, *n* (%):			0.3			>0.9
Sinus	122 (94)	549 (92)		122 (94)	121 (93)	
Atrial fibrillation/flutter	3 (2.3)	36 (6.0)		3 (2.3)	5 (3.8)	
Pacemaker	5 (3.8)	12 (2.0)		5 (3.8)	4 (3.1)	
Cerebral atherosclerosis	5 (3.8)	46 (7.7)	0.2	5 (3.8)	6 (4.6)	>0.9
Peripheral atherosclerosis, *n* (%)	16 (12)	86 (14)	0.7	16 (12)	15 (12)	>0.9
COPD, *n* (%)	13 (10)	80 (13)	0.5	13 (10)	9 (6.9)	>0.9
Stroke, *n* (%)	8 (6.2)	53 (8.8)	0.5	8 (6.2)	5 (3.8)	>0.9
CCS class, *n* (%):			**0.003**			>0.9
I	55 (42)	193 (32)		55 (42)	51 (39)	
II	47 (36)	181 (30)		47 (36)	44 (34)	
III	24 (18)	131 (22)		24 (18)	30 (23)	
IV	4 (3.1)	94 (16)		4 (3.1)	5 (3.8)	
NYHA class, *n* (%):			0.4			>0.9
I	59 (45)	263 (44)		59 (45)	64 (49)	
II	47 (36)	224 (37)		47 (36)	43 (33)	
III	23 (18)	87 (15)		23 (18)	23 (18)	
IV	1 (0.8)	25 (4.2)		1 (0.8)	0 (0)	
History of infarction, *n* (%)	73 (56)	314 (52)	0.6	73 (56)	63 (48)	>0.9
Previous cardiac surgery, *n* (%)	3 (2.3)	49 (8.2)	0.065	3 (2.3)	3 (2.3)	>0.9
Coronary disease, *n* (%):			0.071			0.2
1-vessel disease	77 (59)	286 (48)		77 (59)	64 (49)	
2-vessel disease	27 (21)	195 (33)		27 (21)	46 (35)	
3-vessel disease	26 (20)	118 (20)		26 (20)	20 (15)	
History of PCI, *n* (%)	49 (38)	184 (31)	0.2	49 (38)	45 (35)	>0.9
LVEF (%), median (IQR)	60 (50, 65)	60 (48, 65)	> 0.9	60 (50, 65)	60 (50, 65)	>0.9
Severe heart valve disease, *n* (%)	5 (3.8)	72 (12)	**0.024**	5 (3.8)	4 (3.1)	>0.9
Intravenous nitrates preop, *n* (%)	2 (1.5)	14 (2.3)	0.9	2 (1.5)	2 (1.5)	>0.9
Intravenous inotropes preop, *n* (%)	0 (0)	3 (0.5)	> 0.9	0 (0)	0 (0)	>0.9
Medication—Aspirin, *n* (%)	114 (88)	477 (80)	0.10	114 (88)	112 (86)	>0.9
Medication—other antiagregants, *n* (%)	28 (22)	72 (12)	**0.019**	28 (22)	28 (22)	>0.9
Medication—anticoagulants, *n* (%)	17 (13)	123 (21)	0.12	17 (13)	16 (12)	>0.9
Urgency of surgery, *n* (%):			**0.004**			>0.9
Elective	130 (100)	554 (92)		130 (100)	130 (100)	
Urgent	0 (0)	40 (6.7)		0 (0)	0 (0)	
Emergent	0 (0)	5 (0.8)		0 (0)	0 (0)	
EuroSCORE II (%), median (IQR)	0.93 (0.67, 1.80)	1.18 (0.78, 2.12)	**0.029**	0.93 (0.67, 1.80)	0.90 (0.67, 1.44)	>0.9

BMI, body mass index, cerebral and peripheral atherosclerosis—radiologically proven significant stenosis of corresponding vessels; CCS, Canadian Cardiovascular Society angina classification, Cerebral atherosclerosis—radiologically proven severe stenosis of marginal cerebral arteries; COPD, chronic obstructive pulmonary disease, Emergent surgery—non-elective state when the patient had to be operated on the day of admission; EuroSCORE II, risk prediction model for 30 day post-operative mortality; IQR, interquartile range; LVEF, left-ventricular ejection fraction; NYHA, New York Heart Association dyspnea classification; OAD, oral antidiabetic drugs; PCI, percutaneous coronary intervention; preop, preoperatively, Peripheral atherosclerosis—radiologically proven severe stenosis of limb arteries, Renal failure—patient on dialysis or chronic kidney disease stage 3 or higher according to the National Kidney Foundation, Urgent surgery—non-elective state when the patient had to be operated within one admission for medical reasons. Bold values highlight statistically significant differences according to p-values.

There was no difference in the incidence of perioperative complications among the groups (see Table 2). The RA-MIDCAB group had significantly lower 24-h post-operative blood loss, shorter artificial ventilation time and shorter length of hospital stay.

**Table 2:** Perioperative results pre- and post-matching.

	**Pre-matching**			**Post-matching**		
**Variable**	**Robot-assisted MIDCAB (*n* = 130)**	**Conventional MIDCAB (*n* = 599)**	***p*-value**	**Robot-assisted MIDCAB (*n* = 130)**	**Conventional MIDCAB (*n* = 130)**	***p*-value**
Conversion to sternotomy, *n* (%)	1 (0.8)	14 (2.3)	0.9	1 (0.8)	2 (1.5)	>0.9
Length of surgery (min), median (IQR)	156 (140, 175)	155 (135, 176)	>0.9	156 (140, 175)	155 (135, 180)	>0.9
Reintubation, *n* (%)	1 (0.8)	13 (2.2)	0.6	1 (0.8)	2 (1.6)	>0.9
Artificial ventilation time (hours), median (IQR)	6 (4, 8)	7 (5, 11)	**<0.001**	6 (4, 8)	7 (5, 10)	**0.018**
24 h Blood loss (ml), median (IQR)	300 (200, 450)	450 (300, 638)	**<0.001**	300 (200, 450)	450 (300, 550)	**0.002**
Need of catecholamines >24 h, *n* (%)	34 (26)	212 (35)	0.11	34 (26)	35 (27)	>0.9
Need of inotropes, *n* (%)	4 (3.1)	90 (15)	**0.001**	4 (3.1)	14 (11)	0.083
ICU Length of stay (hours), median (IQR)	23 (19, 30)	24 (21, 46)	0.10	23 (19, 30)	23 (19, 43)	>0.9
Number of transfusions (*n*), median (IQR)	1.00 (1.00, 1.00)	1.00 (1.00, 1.00)	0.5	1.00 (1.00, 1.00)	1.00 (1.00, 1.00)	0.4
Need of transfusions, *n* (%)	12 (9.2)	76 (13)	0.5	12 (9.2)	10 (7.7)	>0.9
Revision for bleeding, *n* (%)	2 (1.5)	19 (3.2)	0.6	2 (1.5)	1 (0.8)	>0.9
Infarction, *n* (%)	1 (0.8)	9 (1.5)	>0.9	1 (0.8)	2 (1.5)	>0.9
Fluidothorax, *n* (%)	26 (20)	160 (27)	0.2	26 (20)	30 (23)	>0.9
Pneumothorax, *n* (%)	0 (0)	17 (2.8)	0.13	0 (0)	1 (0.8)	>0.9
Subcutaneous emphysema, *n* (%)	14 (11)	44 (8.8)	0.6	14 (11)	10 (9.6)	>0.9
Respiraty infection, *n* (%)	2 (1.5)	31 (5.2)	0.2	2 (1.5)	5 (3.8)	>0.9
Oxygenation dysfunction, *n* (%)	5 (3.8)	57 (9.5)	0.10	5 (3.8)	12 (9.2)	0.4
Wound healing complication, *n* (%)	5 (3.8)	25 (4.2)	>0.9	5 (3.8)	2 (1.5)	>0.9
Dialysis, *n* (%)	4 (3.1)	20 (3.3)	>0.9	4 (3.1)	2 (1.5)	>0.9
SIRS, *n* (%)	1 (0.8)	6 (1.0)	>0.9	1 (0.8)	1 (0.8)	>0.9
Post-operative atrial fibrillation, *n* (%)	23 (18)	125 (21)	0.6	23 (18)	22 (17)	>0.9
Length of hospital stay (days), median (IQR)	6.0 (5.0, 7.0)	8.0 (7.0, 10.0)	**<0.001**	6.00 (5.00, 7.00)	8.00 (7.00, 9.00)	**<0.001**
In-hospital mortality, *n* (%)	0 (0)	4 (0.7)	>0.9	0 (0)	0 (0)	>0.9

24 h, 24 hours, Fluidothorax—presence of at least 300 ml of fluid in the pleural cavity according to postoperative sonography; ICU, intensive care unit, Infarction—acute myocardial infarction according to the 4th universal definition of myocardial infarction, Oxygenation dysfunction—hypoxemia in arterial blood gas test post-operatively; SIRS, systemic inflammatory response syndrome. Bold values highlight statistically significant differences according to p-values.

**Figure 2:**
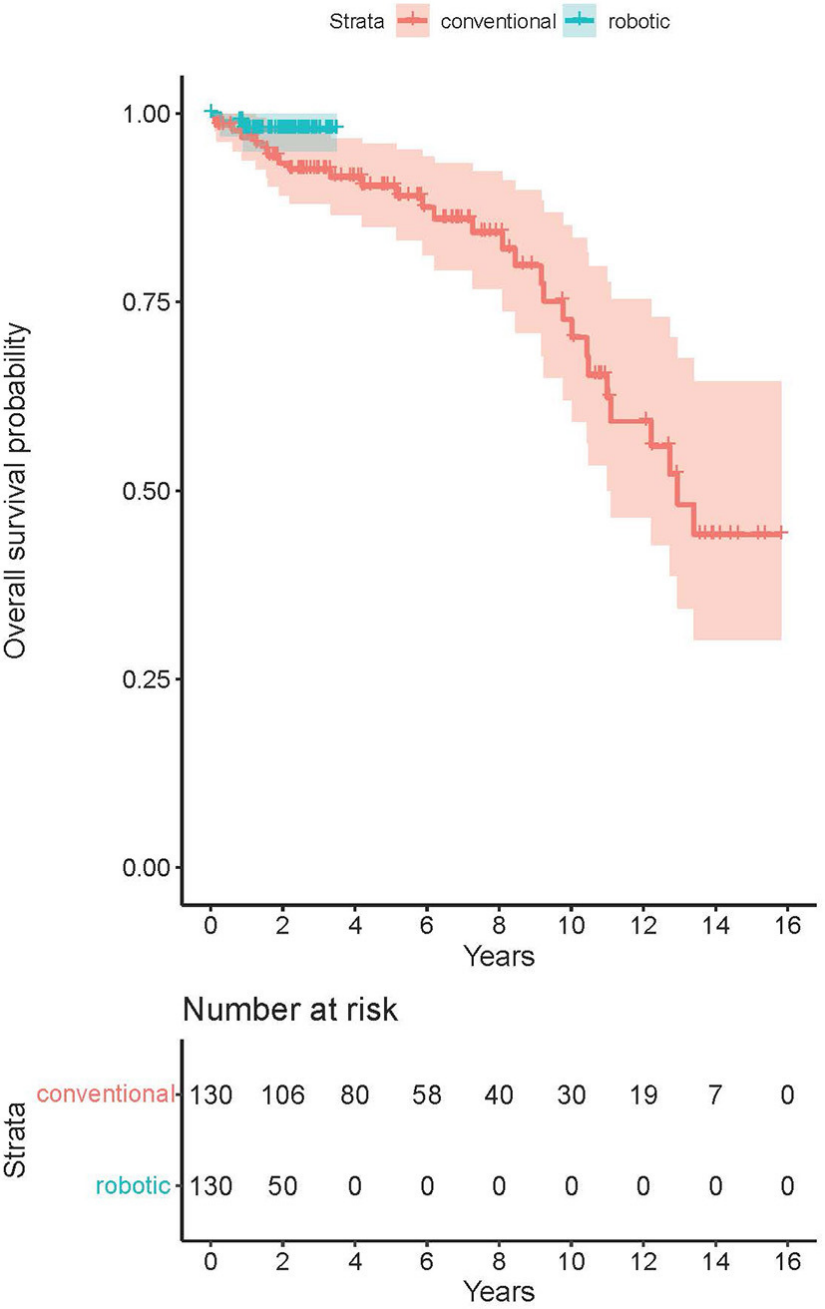
Kaplan-Meier survival analysis of the conventional and robotic MIDCAB groups.

The average post-operative follow-up was 5.6 years in conventional group (up to 15.8 years), and 1.5 years in RA group (up to 3.5 years). There was no difference in all-cause mid-term post-operative mortality among the groups (*p* = 0.14, see Figure 2). The estimated survival according to Kaplan-Meier analysis was 93.5% (CI 89.2–98.0%) at 2 years, 90.4% (CI 85.0–96.0%) at 5 years and 72.7% (CI 62.0–85.2%) at 10 years post-operatively in the MIDCAB group. For the RA-MIDCAB group, only 2-year estimate was performed and was 97.9% (CI 95.0–100.0%).

## Discussion

Despite constant skepticism in the overall surgical community, a rising number of robotic cardiac surgeries and participating centers has been observed since 2015 (15). The technical development of robotic surgery now provides excellent three-dimensional visualization together with enhanced dexterity of robotic arms, being able to perform whole spectrum of procedures: LITA harvest and total robotic revascularization, complex mitral and tricuspid valve surgery, cardiac tumor resections and others (15). The program of robotic cardiac surgery was initiated in 2018 at our department. It has become a part of our long-established minimally invasive cardiac surgery program (MIDCAB since 1997 and minimally invasive mitral and tricuspid valve surgery since 2012). The RA-MIDCAB using the Da Vinci Xi Surgical system (Intuitive, Sunnyvale, CA, USA) has overtaken many of the indications previously referred for conventional MIDCAB which has been a standard of care for 25 years in a selected patient cohort either with single-vessel LAD disease, as a part of hybrid treatment, or even as a palliative revascularization in polymorbid patients with multi-vessel disease.

Although conventional, non-robotic, MIDCAB represents feasible alternative to sternotomy, robotic technique provides high-definition exposure and 3-dimentional telemanipulation that further minimizes the access trauma of the LITA harvest by avoiding larger incision and eliminating rib spreading, dislocation, and fractures. As compared with conventional MIDCAB, the length of the graft is usually longer with the robotic harvesting technology as long as a better access to the proximal and distal part of the graft is facilitated. Robotic telemanipulation allows removal of the pericardial fat, localization of the LAD, and determination of anatomic suitability for minimally invasive approach, as well as optimal intercostal space determination. In addition, RA-MIDCAB can be used for bilateral mammary artery revascularization, a potential benefit in young patients.

To our knowledge, there is no relevant comparison of the two abovementioned techniques in the pertinent literature. Gong et al. compared the conventional MIDCAB with RA-MIDCAB, probably using an older version of da Vinci system (not specified in the article, we assume this due to the fact that study period ended in 2014 when da Vinci Xi was introduced) (13). The outcomes favored RA-MIDCAB with regard to the shorter intensive care unit (ICU) and hospital stay, faster extubation and lower rate of major adverse events in the mid-term despite marginally longer operative duration. However, the data was analyzed retrospectively without any statistical adjustment and could have been biased. Sabashnikov et al. retrospectively compared conventional MIDCAB with RA-MIDCAB and endoscopically assisted MIDCAB adjusted by the propensity score matching (14). With the use of Zeus surgical system (Computer Motion, US) they reported RA-MIDCAB to have the longest operation times. Conventional and RA-MIDCAB had comparable mid-term incidence of angina and major adverse events, which was, however, significantly higher than the endoscopic group. Our study is to date the first to compare conventional MIDCAB with RA-MIDCAB using the newest generation of surgical robotic technology and with proper statistical adjustment to minimize the risk of bias. With regard to a justified suspicion that the “low risk” patients would be forwarded to the RA group, we performed the propensity score matching using a wide scale of preoperative characteristics to select the best match from both consecutive cohorts for the comparison.

Firstly, and most importantly, we report no short-term post-operative mortality in any of the study groups. This is in accordance with the outcomes of conventional MIDCAB from experienced centers (5, 6) as well as with the outcomes of robotic procedures presented by others (13, 16, 17) where the perioperative mortality was consistently below 1%. Similarly, the risk of all post-operative complications was low and equal in both groups.

In discordance with previous evidence, we did not observe longer operation times in the RA group despite including the learning curve. This could be explained by a sophisticated high-quality virtual and dry/wet lab training of the team that must be completed before the start of the robotic program, followed by a support of experienced mentor physically present in the operating room during the first few cases (8, 9).

Interestingly we recorded a lower 24-h post-operative blood loss in the RA group than in the conventional group. This could be explained by three facts: (i) detailed visualization of the LITA harvest leading to a higher comfort during the harvest and securing the side-branches with better precision; (ii) better bleeding control from the LITA bed after taking the vessel down; (iii) smaller skin incision and minimal need for rib spreading required for the distal anastomosis. This evidence could support the choice of RA-MIDCAB over conventional MIDCAB from the technical point of view. On the other hand, the lower blood loss was not clinically significant as it did not lead to a higher risk of re-exploration for bleeding or a higher need for blood transfusions. These were generally low and comparable to the outcomes reported by others (16, 18).

The robotic technology has been long questioned due to higher procedural costs than conventional surgery (19). However, there is some evidence suggesting that the overall treatment costs could be comparable thanks to lower complication rate and shorter length of stay in the ICU and in hospital (10–12, 20, 21). It must be stressed that this evidence stems from the comparison of robotic surgery with full sternotomy approach. In our study, nevertheless, we observed a shorter artificial ventilation time and hospital stay in RA group compared with the technique that is already considered minimally invasive. This could be partially explained by a higher interest toward “fast track” in this group, which could not be eliminated by statistical adjustment. As suggested by Bonatti et al. (22), another objective measures of the speed of recovery in the first post-operative weeks should be addressed in order to better analyze this (such as time needed to return to work, objective exercise tolerance testing etc.). Moreover, future prospective randomized trials with strict criteria for extubation, transfer from the ICU and ideally a fast-track course keeping the principles of the enhanced recovery after surgery (23) may provide us with better insight into post-operative patient reconvalescence.

The long-term post-operative survival in the MIDCAB group (90.4%) was similar to that of Davierwalla et al. (88%) or Reposini et al. (87%) at the 5-year timepoint (6, 7). At the 10-year timepoint, the survival of our group (72.7%) decreased significantly in comparison to the outcomes of Davierwalla et al. (77.7%) or Reposini et al. (84.3%). This significant difference in outcomes could not be explained only by eventual lower life expectancy in Czech Republic than in Germany or Italy. Although the patient's age at the time of surgery was similar in all mentioned analyses [64 in our cohort vs. 64.5 (Davierwalla et al.) vs. 71 years (Reposini et al.)], our cohort yielded a significantly higher proportion of patients with diabetes (33.3 vs. 22.8 vs. 26.1%), history of myocardial infarction (48 vs. 23.8 vs. 5.8%) or previous PCI (35 vs. 21.5 vs. 4.3%), thus suggesting a generally worse long-term prognosis. The analysis of mid-term post-operative survival was obviously limited with regard to the RA group. At this state, the outcomes did not suggest any significant difference among the groups and we assume that the survival will remain comparable to that of conventional MIDCAB.

For less-invasive revascularization options to become widely adopted, both clinical and angiographic outcomes should be comparable to conventional sternotomy approach. Definitive conclusions are not possible without post-operative angiography data, which were unavailable in this retrospective study. However, some studies report excellent patency results. Giambruno et al. published their experience involving patients who underwent RA-MIDCAB procedure with post-procedural graft patency assessment (16). The patency rate of LITA to LAD anastomoses, according to angiography, was 97.4%. Similar results were reported by Halkos et al. with reported 95% LITA patency in patients at the time of discharge after RA-MIDCAB procedure (24). In addition to clinical and surgical benefits, RA-MIDCAB does not compromise the excellent graft patency.

### Limitations

The most important limitation while interpreting the outcomes is the retrospective nature of the study. Despite our maximum effort toward the adjustment of preoperative valuables with propensity-score matching, it is associated with an inherent risk of bias, as for the preoperative patient selection, as for the post-operative patient care.

In this retrospective study we were unable to objectively assess post-operative pain. No strict pain management protocol was utilized in daily routine and a wide scale of additional local/regional infiltration anesthesia strategies have been used over the years.

Finally, the MIDCAB patients were operated over a 15-year period and the RA-MIDCAB patients underwent the surgery mostly in the last 3 years. A constant improvement in perioperative care could eventually lead to relatively worse outcomes of conventional MIDCAB group. Therefore, a shorter artificial ventilation time and hospital stay must be reported with caution. On the other hand, the post-operative blood loss could only hardly be influenced by the recent improvement in perioperative care. We decided to not include the year of surgery as a covariate in the matching for a single reason: most of the patients previously referred for MIDCAB underwent the RA alternative in the last years and the matching could lead to some hidden and unwanted bias in favor of RA-MIDCAB. Moreover, the mortality and the risk of whole spectrum of perioperative complications were generally very low and acceptable in both groups despite the evident limitations.

## Conclusion

The RA-MIDCAB is safe, feasible and attractive alternative to the conventional MIDCAB. It provides the surgeon more comfort during the LITA harvest, it is associated with lower post-operative blood loss and eventually faster rehabilitation after surgery. The risk of death and other post-operative complications is generally very low and comparable in both surgical techniques.

## Data availability statement

The raw data supporting the conclusions of this article will be made available by the authors, without undue reservation.

## Ethics statement

The studies involving human participants were reviewed and approved by Ethics Committee at the University Hospital Hradec Kralove Sokolská 581, 500 05, Hradec Králové, Czechia Reference Number: 202202 P03. Written informed consent for participation was not required for this study in accordance with the national legislation and the institutional requirements.

## Author contributions

JG: substantial contribution to the concept, data collection, data analysis, and drafting of the manuscript. SC, JH, and JV: contribution to the concept, critical revision of the manuscript, and final approval of the version to be published. YS: data collection and linguistic correction. ZS: data collection and critical revision of the manuscript. MV, PS, AS, SM, MK, and JC: data collection. MP: substantial contribution to the concept, data collection, and drafting of the manuscript. All authors contributed to the article and approved the submitted version.

## Funding

This work was supported by the Charles University Research program Cooperatio—Cardiovascular Science.

## Conflict of interest

Author SC has a proctoring contract with Intuitive Surgical. The remaining authors declare that the research was conducted in the absence of any commercial or financial relationships that could be construed as a potential conflict of interest.

## Publisher's note

All claims expressed in this article are solely those of the authors and do not necessarily represent those of their affiliated organizations, or those of the publisher, the editors and the reviewers. Any product that may be evaluated in this article, or claim that may be made by its manufacturer, is not guaranteed or endorsed by the publisher.

## Abbreviations

CCS: Canadian Cardiovascular Socienty chest angina classification
LAD: left anterior descending artery
LITA: left internal thoracic artery
MIDCAB: minimally invasive direct coronary artery bypass grafting
NYHA: New York Heart Association dyspnea classification
RA: robotically assisted
TECAB: totally endoscopic coronary artery bypass grafting.
